# IFN-γ-induced increase in the mobility of MHC class II compartments in astrocytes depends on intermediate filaments

**DOI:** 10.1186/1742-2094-9-144

**Published:** 2012-06-26

**Authors:** Nina Vardjan, Mateja Gabrijel, Maja Potokar, Urban Švajger, Marko Kreft, Matjaž Jeras, Yolanda de Pablo, Maryam Faiz, Milos Pekny, Robert Zorec

**Affiliations:** 1 Celica Biomedical Center, Tehnološki park 24, Ljubljana 1000, Slovenia; 2 Laboratory of Neuroendocrinology-Molecular Cell Physiology, Institute of Pathophysiology, Faculty of Medicine, University of Ljubljana, Zaloška 4, Ljubljana 1000, Slovenia; 3 Blood Transfusion Center of Slovenia, Šlajmerjeva 6, Ljubljana 1000, Slovenia; 4 Biotechnical Faculty, University of Ljubljana, Večna pot 111, Ljubljana 1000, Slovenia; 5 Faculty of Pharmacy, University of Ljubljana, Aškerčeva cesta 5, Ljubljana 1000, Slovenia; 6 Center for Brain Repair and Rehabilitation, Department of Clinical Neuroscience and Rehabilitation, Institute of Neuroscience and Physiology, Sahlgrenska Academy at University of Gothenburg, Medicinaregatan 9A, Gothenburg, 413 90, Sweden

**Keywords:** Astrocytes, Vesicle mobility, Late endosomes/lysosomes, Major histocompatibility class II compartments, Interferon-γ, Dextran labeling, Immune response

## Abstract

**Background:**

In immune-mediated diseases of the central nervous system, astrocytes exposed to interferon-γ (IFN-γ) can express major histocompatibility complex (MHC) class II molecules and antigens on their surface. MHC class II molecules are thought to be delivered to the cell surface by membrane-bound vesicles. However, the characteristics and dynamics of this vesicular traffic are unclear, particularly in reactive astrocytes, which overexpress intermediate filament (IF) proteins that may affect trafficking. The aim of this study was to determine the mobility of MHC class II vesicles in wild-type (WT) astrocytes and in astrocytes devoid of IFs.

**Methods:**

The identity of MHC class II compartments in WT and IF-deficient astrocytes 48 h after IFN-γ activation was determined immunocytochemically by using confocal microscopy. Time-lapse confocal imaging and Alexa Fluor^546^-dextran labeling of late endosomes/lysosomes in IFN-γ treated cells was used to characterize the motion of MHC class II vesicles. The mobility of vesicles was analyzed using ParticleTR software.

**Results:**

Confocal imaging of primary cultures of WT and IF-deficient astrocytes revealed IFN-γ induced MHC class II expression in late endosomes/lysosomes, which were specifically labeled with Alexa Fluor^546^-conjugated dextran. Live imaging revealed faster movement of dextran-positive vesicles in IFN-γ-treated than in untreated astrocytes. Vesicle mobility was lower in IFN-γ-treated IF-deficient astrocytes than in WT astrocytes. Thus, the IFN-γ-induced increase in the mobility of MHC class II compartments is IF-dependent.

**Conclusions:**

Since reactivity of astrocytes is a hallmark of many CNS pathologies, it is likely that the up-regulation of IFs under such conditions allows a faster and therefore a more efficient delivery of MHC class II molecules to the cell surface. In vivo, such regulatory mechanisms may enable antigen-presenting reactive astrocytes to respond rapidly and in a controlled manner to CNS inflammation.

## Background

Antigen-presenting cells (APCs) are key players in the immune response. They take up and process exogenous antigens and present them to CD4 helper T-cells, resulting in antigen-specific T-cell activation. After endocytosis of antigens by APCs, early endosomes gradually transform into late endosomes/lysosomes, where antigens are processed to peptides and loaded onto MHC class II molecules. Late endosomes/lysosomes expressing peptide-MHC class II complexes are delivered to the cell surface for recognition by T-cell receptors on CD4 helper T-cells [1,2].

Astrocytes, the most abundant glial cells in the central nervous system (CNS), can act as ‘non-professional’ APCs. Unlike professional APCs (for example, dendritic cells, macrophages, B-cells), astrocytes express cell-surface MHC class II molecules only upon exposure to the cytokine IFN-γ [3,4]. IFN-γ-activated astrocytes participate in antigen presentation and activation of CD4 helper T-cells in immune-mediated CNS disorders such as multiple sclerosis [5,6] and in experimental autoimmune encephalomyelitis [3]. Expression of MHC II molecules in astrocytes is thought to be controlled by neurons, since IFN-γ induces MHC class II expression only in slice culture zones containing degenerated neurons, in which spontaneous neuronal activity (for example, release of glutamate or noradrenaline) is suppressed [7,8]. In primary astrocyte cultures without neurons, IFN-γ induces expression of surface MHC class II and some APC-specific co-stimulatory molecules [9,10]. Such cultures may be useful for studying antigen presentation at the subcellular level.

The trafficking of MHC class II compartments in APCs is dependent on the cytoskeletal network. This trafficking is influenced by actin microfilaments [11] and actin-based motor proteins in B-cells [12] and by microtubules and microtubule-based motor proteins in dendritic cells and a human melanoma cell line [13,14]. The MHC class II compartments in astrocytes and their mobility have not been characterized.

In reactive astrocytes, which are found in almost all CNS pathologies, the expression of IF proteins (cell-type-specific components of the cytoskeleton) [15] is up-regulated [16,17]. Studies of primary astrocytes from mice deficient in the IF proteins glial fibrillary acidic protein and vimentin (*GFAP*^*-/-*^*Vim*^*-/-*^) and completely devoid of astrocyte cytosolic IFs [18,19] have suggested that the up-regulation of IFs in pathological situations may deregulate vesicle trafficking [19,20].

In this study, we sought to determine whether the trafficking of MHC class II compartments, which is closely associated with neuroinflammation, involves IFs. Live imaging revealed faster movement of dextran-positive vesicles in IFN-γ-treated than in untreated astrocytes. These compartments enriched with MHC class II molecules upon IFN-γ treatment, were less mobile in cytosolic IF-deficient (*GFAP*^*-/-*^*Vim*^*-/-*^) than in WT astrocytes, indicating that the IFN-γ-induced increase in the mobility of MHC class II compartments in astrocytes depends on IFs.

## Methods

### Cell cultures

Astrocyte cultures were prepared from the cerebrum of 1-day-old *GFAP*^*-/-*^*Vim*^*-/-*^ and WT mice on a mixed C57Bl6/129Sv/129Ola genetic background and maintained as described [18,21-23]. *GFAP*^*-/-*^*Vim*^*-/-*^ mice [18,24] were obtained by cross-breeding mice lacking GFAP [22] and mice lacking Vim [25]. Before experiments, the cells were removed from the culture flasks with trypsin**/**EDTA and plated on 22-mm glass coverslips coated with poly-L-lysine. Cells were maintained in high-glucose Dulbecco’s modified Eagle’s medium supplemented with 10% fetal bovine serum, 1 mM sodium pyruvate, 2 mM L-glutamine, and 25 μg/mL penicillin-streptomycin in an atmosphere of humidified air (95%) and CO_2_ (5%). In some experiments, astrocytes were treated with 600 U/ml IFN-γ (0.06 μg/mL; Abcam, Cambridge, UK) for 48 h at 37°C. The purity of astrocyte cultures was confirmed with antibodies against astrocytic markers GFAP (Sigma Aldrich, St Louis, MO, USA) or glutamine synthetase (Abcam, Cambridge, MA, USA). All chemicals were from Sigma Aldrich (St Louis, MO, USA) unless noted otherwise.

The care for experimental animals was in accordance with International Guiding Principles for Biomedical Research Involving Animals developed by the Council for International Organizations of Medical Sciences and Directive on Conditions for issue of License for Animal Experiments for Scientific Research Purposes (Official Gazette of the RS, No. 43/07).

### Flow cytometry

Cell-surface expression of MHC class II molecules was determined by flow cytometry (FACSCalibur, BD Biosciences, Franklin Lakes, NJ, USA) and fluorescence-labeled rat Alexa Fluor^488^ anti-mouse I-A/I-E (MHC class II) antibodies (BioLegend, San Diego, CA, USA). Control and IFN-γ-activated mouse WT and *GFAP*^*-/-*^*Vim*^*-/-*^ astrocytes were removed from flasks with trypsin/EDTA and collected by centrifugation. Antibody was added, and the cells were incubated at room temperature for 15 min in the dark, washed twice with Dulbecco’s phosphate-buffered saline, and resuspended in 2% paraformaldehyde. Alexa Fluor^488^-IgG2b cocktail (BioLegend) was used as an isotype control. From a forward scatter and side scatter dot plot**,** the population of cells was gated for further analysis. Cell-surface expression of MHC class II molecules was determined by fluorescence-activated cell sorting.

### Immunostaining

Control and IFN-γ-activated astrocytes growing on coverslips were fixed in 2% paraformaldehyde for 5 min at room temperature and treated with 10% goat serum for 1 h at 37°C. In some experiments, astrocytes were incubated with fixable 10-kDa Alexa Fluor^546^ dextran conjugate (0.1 mg/mL; Molecular Probes, Invitrogen, Eugene, OR, USA) for 16 h at 37°C and washed for 3 h at 37°C. Cultures were then stained with one or both of the following primary antibodies for 2 h at 37°C: Alexa Fluor^488^ rat anti-mouse MHC class II (1:200) and rabbit anti-lysosomal-associated membrane protein 1 **(**LAMP1; 1:250; Abcam). Excess primary antibody was washed off, and the cultures were incubated for 1 h at 37°C with Alexa Fluor^546^- or Alexa Fluor^488^-conjugated secondary goat anti-rabbit IgG antibody (1:600; Abcam). Excess antibody was removed, and cells were treated with SlowFade Gold antifade reagent (Molecular Probes). Immunolabeled cells were imaged with an inverted Zeiss LSM 510 or Zeiss LSM 510 Meta confocal microscopes with an oil-immersion plan apochromatic objective (63x, 1.4 NA; Carl Zeiss, Jena, Germany), using 488-nm Ar-Ion and 543-nm He-Ne laser excitation. Emission light was acquired sequentially with a 505-530-nm bandpass emission filter (Alexa Fluor^488^) and a 560-nm long-pass emission filter (Alexa Fluor^546^).

### Time-lapse confocal imaging

Astrocytes were loaded with 0.1 mg/mL Alexa Fluor^546^-dextran (Molecular Probes) for 16 h at 37°C, washed for 3 h at 37°C, and transferred to the chamber for imaging. Time-lapse fluorescence images (512 x 512 pixels) were obtained every 2 s for 3 min (90 frames) with the Zeiss LSM 510 Meta confocal microscope described above and 543-nm He-Ne laser excitation. Emission light was acquired with a 560-nm long-pass emission filter. Sixty seconds after the start of recording cells were superfused with 1 mM ATP in a standard saline solution (10 mM Hepes/NaOH, pH 7.2, 10 mM D-glucose, 131.8 mM NaCl, 1.8 mM CaCl_2_, 2 mM MgCl_2_, and 5 mM KCl).

### Vesicle mobility

The motion of Alexa Fluor^546^-dextran–labeled vesicles before and 30 s after the start of ATP stimulation was tracked in exported TIFF files by ParticleTR software (Celica, Ljubljana, Slovenia) as described [23]. A single dextran-labeled vesicle was selected in the image sequence. Using a simplex algorithm with a least-squares estimator, the software fits a 2D Gaussian curve to the vesicle intensity profile. The peak of curve was recorded as the *x,y* coordinates of the vesicle position. The time (from the beginning of tracking for a single vesicle), step length (displacement of a vesicle over 2 s), TL (total length of the analyzed vesicle pathway), and MD (measure of a net translocation of vesicle) were determined [19,23]. Vesicle mobility in control and IFN-γ-activated astrocytes before and after ATP stimulation was analyzed in 40 cells from five independent mouse WT astrocyte cultures and in 33 cells from four independent mouse *GFAP*^*-/-*^*Vim*^*-/-*^ astrocyte cultures. Vesicle mobility was analyzed for 30 s (15 frames).

### Statistical analysis

Data are presented as mean ± s.e.m. Differences in TL and MD were analyzed by *t* test. Differences in the fraction of vesicles exhibiting directional mobility were analyzed with Fisher’s exact test. Differences between the slopes were analyzed by Analysis of covariance. *P* <0.05 was considered significant.

## Results

### IFN-γ induces expression of MHC class II molecules on *GFAP*^*-/-*^*Vim*^*-/-*^ astrocytes

**Figure 1:**
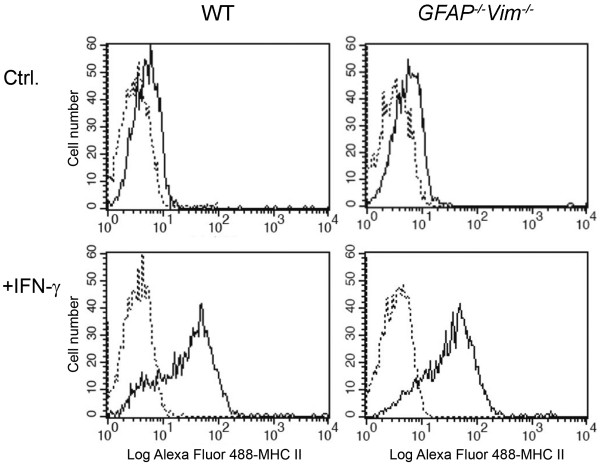
**IFN-γ induces MHC class II expression on WT and*GFAP***^***-/-***^***Vim***^***-/-***^** deficient mouse astrocytes.** Cell-surface expression of MHC class II molecules on WT (left) and *GFAP*^*-/-*^*Vim*^*-/-*^ (right) astrocytes was analyzed by flow cytometry in the absence (Ctrl.) and 48 h after the addition of 600 U/ml IFN-γ (+IFN-γ). Note that in WT cells, the fraction of low fluorescence intensity MHC class II positive cells upon IFN-γ treatment appears to be higher than in IFN-γ treated *GFAP*^*-/-*^*Vim*^*-/-*^ astrocytes. Dotted lines indicate isotype controls; solid lines indicate anti-Alexa Fluor^488^-MHC class II antibodies. The results are representatives of four independent experiments.

To determine whether MHC class II molecules are expressed on the surface of IF-deficient astrocytes, we exposed primary cultures of WT and *GFAP*^*-/-*^*Vim*^*-/-*^ astrocytes to IFN-γ for 48 h. Cell-surface expression of MHC class II molecules was much more abundant in control WT and *GFAP*^*-/-*^*Vim*^*-/-*^ cells treated with IFN-γ than in non-treated cells (Figure 1). Although the fraction of low fluorescence intensity MHC class II positive cells upon IFN-γ treatment was slightly higher than in IFN-γ treated *GFAP*^*-/-*^*Vim*^*-/-*^ astrocytes (Figure 1, lower panels), IFs are not required for expression of MHC class II molecules on the surface of astrocytes.

### Fluorescent dextran labels MHC class II–positive late endosomes/lysosomes in WT and *GFAP*^*-/-*^*Vim*^*-/-*^ astrocytes

**Figure 2:**
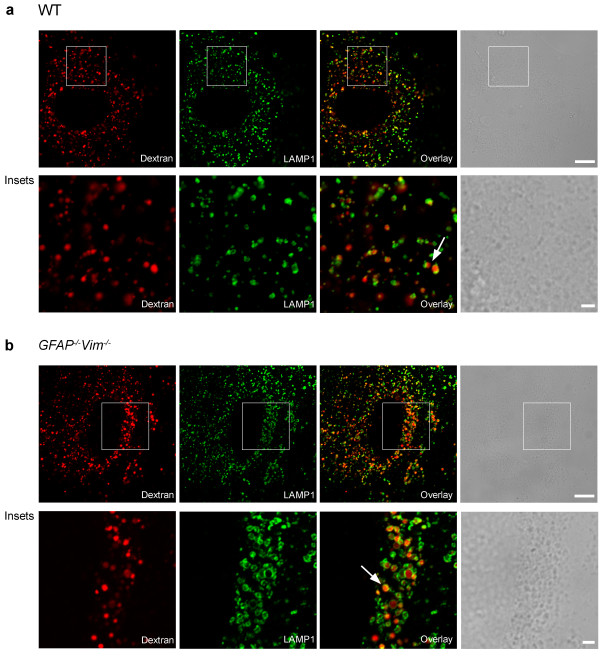
**Alexa Fluor**^**546**^**-dextran labels late endosomes/lysosomes in WT and*GFAP***^***-/-***^***Vim***^***-/-***^** mouse astrocytes.** Fluorescence images of WT (**a**) and *GFAP*^*-/-*^*Vim*^*-/-*^ (**b**) astrocytes labeled with dextran and immunostained with antibodies against LAMP1, a marker of late endosomes/lysosomes. Merged images (overlay) show that the majority of dextran puncta are colocalized with LAMP1 puncta. Lower panels (insets) show boxed regions at higher magnification. Scale bars: 10 μm, 2 μm (insets). Arrowheads point to typical structures expressing both signals. Note that the green signal corresponding to the membrane bound LAMP-1 signal encircles the luminal signal of red dextran.

Before reaching the cell surface of APCs, MHC class II molecules are generally located in late endosomal/lysosomal compartments of APCs [1] with diameters above 200 nm [26,27], which can be specifically labeled by long-term loading of cells with fluorescent dextrans [28]. We used this approach to label and study the mobility of late endosomes/lysosomes expressing MHC class II molecules in IFN-γ-activated WT and IF*-*deficient astrocytes. First, we determined whether dextran labels late endosomes/lysosomes in WT and *GFAP*^*-/-*^*Vim*^*-/-*^ astrocytes and whether these compartments express MHC class II molecules upon IFN-γ stimulation. The cells were preloaded with Alexa Fluor^546^-dextran for 16 h and washed with dextran-free medium for 3 h to clear dextran from the early endosomal compartments [28,29]. WT and *GFAP*^*-/-*^*Vim*^*-/-*^ astrocytes internalized dextran without any stimulation (Figure 2), consistent with previous studies of the endocytosis of dextrans [28,30] and FM-dyes [31,32] in astrocytes. Fluorescent dextran was found in punctate structures in the perinuclear region and throughout the cytoplasm.

To verify that the dextran-labeled structures were late endosomes/lysosomes, we fixed and stained the cells with antibodies against LAMP1, a marker of late endosomes/lysosomes. In both WT and *GFAP*^*-/-*^*Vim*^*-/-*^ astrocytes, internalized Alexa Fluor^546^-dextran was highly colocalized with LAMP1 (Figure 2). Thus, the lack of IFs in *GFAP*^*-/-*^*Vim*^*-/-*^ astrocytes does not affect long-term dextran loading into late endosomes/lysosomes.

**Figure 3:**
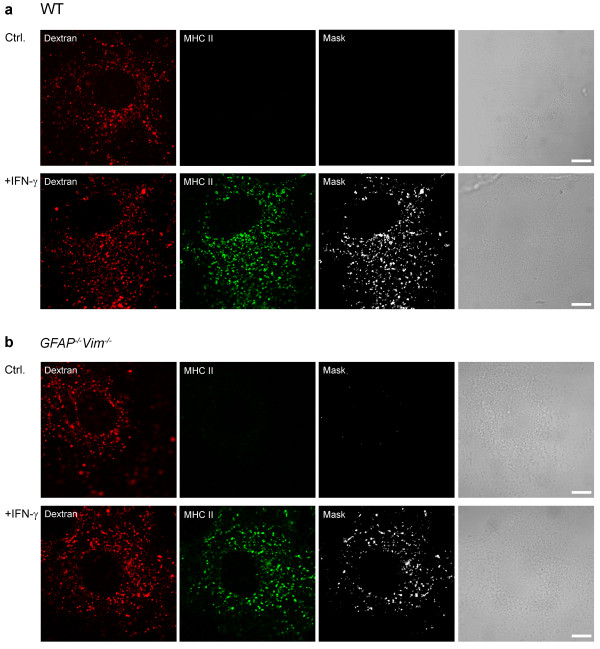
**Alexa Fluor**^**546**^**-dextran labels MHC class II–positive compartments in IFN-γ-treated WT and*GFAP***^***-/-***^***Vim***^***-/-***^** mouse astrocytes.** Fluorescence images of control (Ctrl.) and IFN-γ-treated (+IFN-γ) WT (**a**) and *GFAP*^*-/-*^*Vim*^*-/-*^ (**b**) astrocytes labeled with dextran, fixed, and immunostained with antibodies against MHC class II molecules. IFN-γ induces punctuate expression of MHC class II molecules. White pixels (mask) represent the colocalization mask of green (MHC II) and red fluorescence pixels (dextran). Note that the threshold value of 51 arbitrary units of 255 intensity levels, which corresponds to 20% of the maximum intensity level, was selected to separate the background intensity levels from the signal of single red and green pixels. Scale bars: 10 μm.

**Figure 4:**
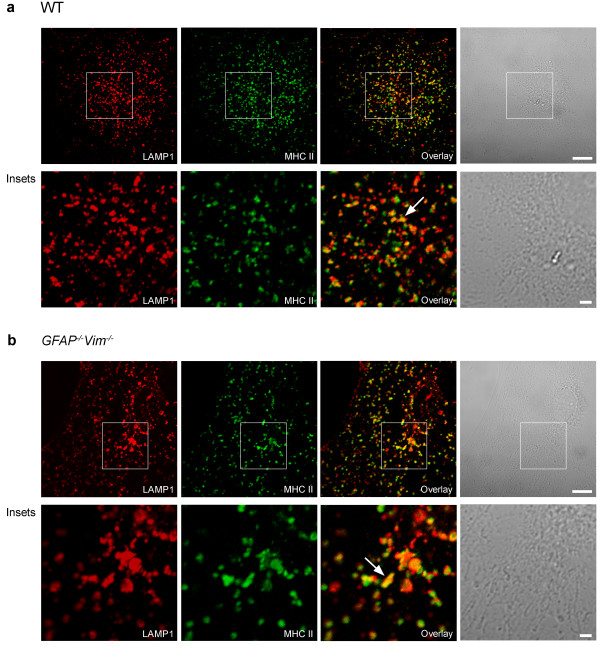
**MHC class II molecules are expressed in late endosomes/lysosomes in IFN-γ-activated WT and*GFAP***^***-/-***^***Vim***^***-/-***^**mouse astrocytes.** Fluorescence images of IFN-γ-activated WT (**a**) and *GFAP*^*-/-*^*Vim*^*-/-*^ (**b**) astrocytes after immunostaining with antibodies against LAMP1 and MHC class II molecules. The two signals overlap to a great extent (overlay), indicating that IFN-γ induces expression and localization of MHC class II molecules in late endosomes/lysosomes in WT and *GFAP*^*-/-*^*Vim*^*-/-*^ astrocytes. Lower panels (insets) show boxed regions at higher magnification. Scale bars: 10 μm; 2 μm (insets). Arrowheads point to typical structures expressing both signals.

Next we examined whether dextran-labeled late endosomes/lysosomes express MHC class II molecules when treated with IFN-γ. After a 48-h exposure to IFN-γ, cells were labeled with Alexa Fluor^546^-dextran and antibodies against MHC class II molecules. MHC class II molecules were present in vesicles in treated *GFAP*^*-/-*^*Vim*^*-/-*^ and WT cells but not in untreated cells (Figure 3). Internalized Alexa Fluor^546^-dextran was highly colocalized with MHC class II molecules (Figure 3a, b, lower panels), indicating that dextran was transported into the MHC class II-enriched compartments. To further characterize these compartments, we double labeled IFN-γ-activated cells with antibodies against MHC class II molecules and LAMP1. MHC class II molecules colocalized with LAMP1 in WT and *GFAP*^*-/-*^*Vim*^*-/-*^ astrocytes stimulated with IFN-γ (Figure 4).

### Increased mobility of dextran-labeled vesicles in IFN-γ-activated astrocytes requires IFs

Next we used dextran labeling and time-lapse confocal imaging to study the mobility of late endosomes/lysosomes in living astrocytes. To determine whether vesicle mobility in IFN-γ-treated cells is dependent on cytosolic Ca^2+^ levels, we exposed the cells to 1 mM ATP to increase cytosolic [Ca^2+^[33,34]. Cells were imaged every 2 s for 30 s (15 frames) to determine the maximal displacement (MD) and the track length (TL) of vesicles.

**Figure 5:**
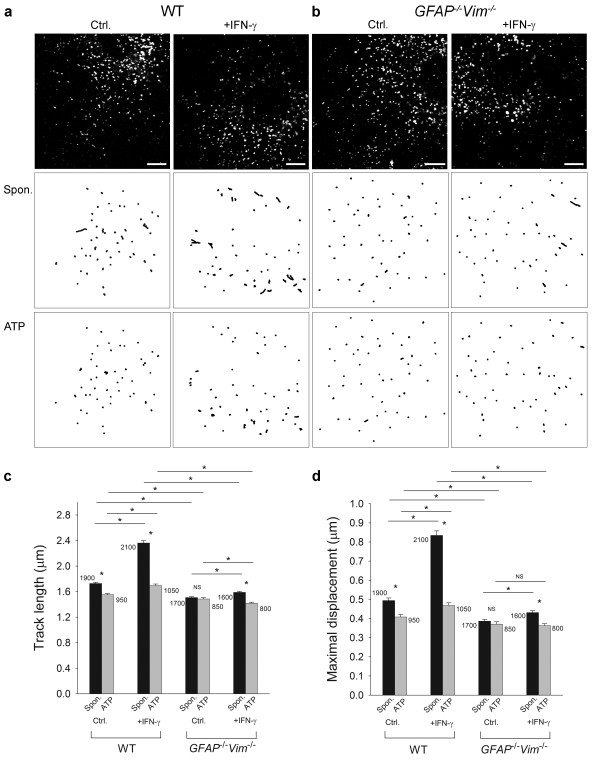
**IFN-γ induces a larger increase in the mobility of dextran labeled vesicles in WT than in*GFAP***^***-/-***^***Vim***^***-/-***^**mouse astrocytes.** (**a, b**) Top, representative fluorescence images of a control (Ctrl.) and an IFN-γ-treated (+IFN-γ) WT astrocyte (**a**) and a *GFAP*^*-/-*^*Vim*^*-/-*^ astrocyte (**b**) labeled with dextran. Bottom, trajectories of dextran-loaded vesicles in an exemplary cell before (Spon.) and after treatment with 1 mM ATP (ATP). The tracks of individual vesicles were monitored for 30 s (see also the movies, Additional files 1, 2, 3, 4, 5, 6, 7, 8). Vesicle tracks were longer in WT and *GFAP*^*-/-*^*Vim*^*-/-*^ cells treated with IFN-γ than in untreated cells. ATP decreased vesicle mobility. Scale bars: 10 *μ*m. (**c**) Histogram of average vesicle TLs in control (Ctrl.) and IFN-γ-treated (+IFN-γ) WT and *GFAP*^*-/-*^*Vim*^*-/-*^ cells. (**d**) Histogram of mean MDs of vesicles in control (Ctrl.) and IFN-γ-treated (+IFN-γ) WT and *GFAP*^*-/-*^*Vim*^*-/-*^ cells. Numbers on graphs are numbers of analyzed vesicles. Values are mean ± s.e.m. *P <0.05.

Dextran-labeled vesicles moved in the cytoplasm of WT astrocytes (Figure 5a; see also the movies Additional files 1,2) [32]. In IFN-γ-stimulated cells, where the majority of dextran-labeled vesicles were enriched with MHC class II molecules (Figure 3), the vesicle tracks were longer than in control cells. TL increased by 36.4% (*P* <0.05) and MD by 69.4% (*P* <0.05) (Figure 5c, d, Table 1). Thus, IFN-γ increases the mobility of dextran-labeled vesicles. In *GFAP*^*-/-*^*Vim*^*-/-*^ astrocytes, dextran-labeled vesicles continued to move (Figure 5b, see also the movies Additional files 3,4) but did so more slowly than in WT astrocytes, and the IFN-γ-induced increase in mobility was smaller than in WT astrocytes. TL increased by 5.3% and MD by 10.3% (*P* <0.05) (Figure 5c, d, Table 1).

**Table 1:** **Mobility of vesicles in control and IFN-γ-treated WT and*GFAP***^*-/-*^***Vim***^*-/-*^**astrocytes under spontaneous conditions and after ATP stimulation**

**Genotype**	**Stimulation**	**Control**			**IFN-γ**		
		**TL (μm)**	**MD (μm)**	***n***	**TL (μm)**	**MD (μm)**	***n***
WT	Spontaneous	1.73 ± 0.02	0.49 ± 0.01	1900	2.36 ± 0.04^a^	0.83 ± 0.02^a^	2100
	ATP	1.55 ± 0.02^b^	0.41 ± 0.01^b^	950	1.70 ± 0.02^b^	0.47 ± 0.01^b^	1050
*GFAP*^*-/-*^*Vim*^*-/-*^	Spontaneous	1.51 ± 0.01	0.39 ± 0.01	1700	1.59 ± 0.02^a^	0.43 ± 0.01^a^	1600
	ATP	1.49 ± 0.02	0.37 ± 0.01	850	1.42 ± 0.02^b^	0.36 ± 0.01^b^	800

Values are mean ± s.e.m.

*n*, number of vesicles.

^a^*P* < 0.05 *vs.* controls.

^b^*P* < 0.05 *vs.* spontaneous conditions.

After ATP stimulation (Figure 5, Table 1, see also the movies Additional files 5, 6), the mobility of dextran-labeled vesicles was reduced in control and IFN-γ-treated WT astrocytes. In control WT cells, TL decreased by 10.4% and MD by 16.3% (*P* <0.05). In IFN-γ-treated WT astrocytes, the decreases were larger. TL decreased by 28.0% and MD by 43.4% (*P* <0.05). Thus, the mobility of dextran-labeled vesicles in control and in IFN-γ-activated WT astrocytes depends on cytosolic [Ca^2+^].

Stimulation with ATP did not reduce the mobility of dextran-labeled vesicles in control *GFAP*^*-/-*^*Vim*^*-/-*^ astrocytes but did in IFN-γ-activated *GFAP*^*-/-*^*Vim*^*-/-*^ astrocytes (Figure 5, Table 1; see also the movies Additional files 7, 8). TL was reduced by 10.7% and MD by 16.3% (*P* <0.05).

### IFN-γ increases the proportion of directional vesicles in WT astrocytes

**Figure 6:**
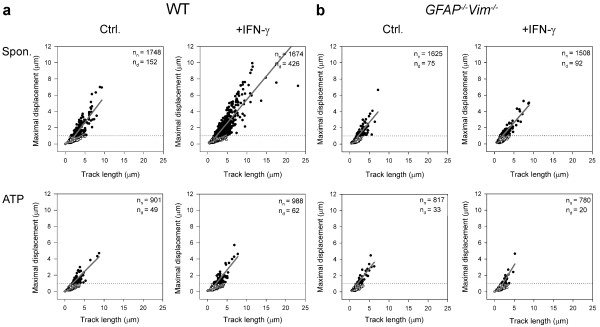
**IFN-γ increases the proportion of directional dextran labeled vesicles in live WT mouse astrocytes.** Graph shows MD as a function of total TL over 30 s. Vesicles with MD >1 μm (dashed line) were characterized as directional vesicles (black circles; n_d_, number of directional vesicles). Other vesicles were characterized as non-directional vesicles (white circles; n_n_, number of nondirectional vesicles). (**a**) The percentage of directional vesicles in IFN-γ-treated WT cells was greater than in untreated control cells (20.3% *vs.* 8%; *P* <0.001). After ATP treatment, the percentage of directional vesicles decreased from 8% to 5.2% (*P* <0.01) in control cells and from 20.3% to 5.9% in IFN-γ-treated WT cells (*P* <0.001). (**b**) The percentage of directional vesicles in IFN-γ-treated *GFAP*^*-/-*^*Vim*^*-/-*^ astrocytes did not change significantly compared to untreated control cells (4.4% *vs.* 5.7%, *P* = 0.081). After ATP treatment, the percentage of directional vesicles was not affected in control cells (4.4% *vs.* 3.9%; *P* = 0.602), but was reduced from 5.7% to 2.5% in IFN-γ-treated *GFAP*^*-/-*^*Vim*^*-/-*^ cells (*P* <0.001). Note that the slopes of the lines fitted for directional and non-directional vesicles are presented in Table 2.

Next we analyzed the effect of IFN-γ on the directional mobility of vesicles. Dextran-labeled vesicles in WT and *GFAP*^*-/-*^*Vim*^*-/-*^ astrocytes exhibited two types of mobility (Figure 6): directional (MD >1 μm) and non-directional (MD <1 μm) [19,23]. In WT and *GFAP*^*-/-*^*Vim*^*-/-*^ astrocytes, the slopes of regression lines for mobility data of directional and non-directional vesicles differed before and after ATP treatment (*P* <0.001, Figure 6, Table 2). Treatment with IFN-γ increased the proportion of vesicles with directional mobility in WT astrocytes (from 8.0% to 20.3%, *P* <0.001) but not in *GFAP*^*-/-*^*Vim*^*-/-*^ astrocytes (4.4% *vs.* 5.7%, *P* = 0.081) (Figure 6), suggesting that the effect of IFN-γ on directional mobility of vesicles involves IFs.

**Table 2:** **Percentage of directional and non-directional vesicles before and after ATP treatment for control and IFN-γ-treated WT and*GFAP***^***-/-***^***Vim***^***-/-***^**astrocytes and slopes of the lines fitted to the vesicle mobility data**

**Genotype**	**Stimulation**	**Control**		**IFN-γ**	
	**Mobility**	**Slope (a ± s.e.m.)**^**a**^	**Vesicle**	**Slope (a ± s.e.m.)**^**a**^	**Vesicle**
			**(%)**		**(%)**
WT	Spontaneous				
	Directional	0.633 ± 0.038^c^ (152)^b^	8.0	0.575 ± 0.020^c^ (426)	20.3
	Non-directional	0.262 ± 0.006 (1748)	92.0	0.219 ± 0.005 (1674)	79.7
	ATP				
	Directional	0.483 ± 0.056^c^ (49)	5.2	0.597 ± 0.059^c^ (62)	5.9
	Non-directional	0.266 ± 0.009 (901)	94.8	0.231 ± 0.007 (988)	94.1
*GFAP*^*-/-*^*Vim*^*-/-*^	Spontaneous				
	Directional	0.581 ± 0.068^c^ (75)	4.4	0.553 ± 0.030^c^ (92)	5.7
	Non-directional	0.324 ± 0.008 (1625)	95.6	0.329 ± 0.009 (1508)	94.3
	ATP				
	Directional	0.616 ± 0.069^c^ (33)	3.9	0.781 ± 0.148^c^ (20)	2.5
	Non-directional	0.298 ± 0.010 (817)	96.1	0.279 ± 0.012 (780)	97.5

^a^The slope values (a) ± s.e.m were obtained from the linear function fitted to the mobility data in Figure 6.

^b^Values inside brackets are numbers of vesicles. The slopes of regression lines for mobility data of directional and nondirectional vesicles differed in control and IFN-γ treated WT and *GFAP*^*-/-*^*Vim*^*-/-*^ cells under spontaneous conditions and after ATP treatment.

^c^*P* <0.001 *vs.* non-directional mobility.

The fraction of vesicles with directional mobility was smaller in control and IFN-γ-treated *GFAP*^*-/-*^*Vim*^*-/-*^ astrocytes than in control and IFN-γ-treated WT astrocytes (4.4% *vs.* 8.0% and 5.7% *vs.* 20.3%, respectively; *P* <0.001) (Figure 6, upper panels), indicating that IFs affect the directional mobility of vesicles in control [19,23] and IFN-γ-treated astrocytes.

Stimulation with ATP decreased the percentage of directionally mobile dextran-labeled vesicles in control (8.0% *vs.* 5.2%, *P* <0.01) and IFN-γ-treated WT astrocytes (20.3% *vs.* 5.9%, *P* <0.001) (Figure 6a), as well as in control (4.4% *vs.* 3.9%, P = 0.602) and IFN-γ-treated *GFAP*^*-/-*^*Vim*^*-/-*^ astrocytes (5.7% *vs.* 2.5%, *P* <0.001) (Figure 6b).

## Discussion

This study shows that IFN-γ induces an IF-dependent increase in the mobility of MHC class II compartments in astrocytes. Our findings suggest that up-regulation of IFs in reactive astrocytes allows faster and therefore more efficient delivery of MHC class II molecules to the cell surface.

### The expression and targeting of MHC class II molecules in IFN-γ-activated astrocytes

In WT and *GFAP*^*-/-*^*Vim*^*-/-*^ primary mouse astrocytes, IFN-γ induced expression of MHC class II molecules on the cell surface and in vesicular structures. As in professional APCs [1], MHC class II expression colocalized with dextran and LAMP1, which are markers of late endosomes/lysosomes. Thus, although IFs are the key structural and functional element in reactive astrocytes [16,24,35] - and may participate in protein targeting defects in polarized epithelial cells [15], lymphocytes and endothelial cells [36], dorsal root ganglion neurons [37], and fibroblasts [38] - the lack of IFs in astrocytes does not affect the distribution of late endosomal/lysosomal markers or MHC class II expression in these organelles 48 h after IFN-γ activation. Interestingly, the distribution of the late endosomal/lysosomal markers LAMP1 and CD63 is altered in *Vim*^*-/-*^ fibroblasts [38].

### IFN-γ induces an IF-dependent increase in the mobility of MHC class II compartments in astrocytes

IFs can deregulate trafficking of vesicular glutamate transporter 1, atrial natriuretic peptide, and acidic vesicles in astrocytes [19,20] and participate in the transport and distribution of mitochondria in neurons [39], Golgi complexes in COS-7 cells [40], late endosomes/lysosomes in fibroblasts [38], and melanosomes in melanophores [41]. To study the effect of IFs on the trafficking of MHC class II molecules in IFN-γ-activated astrocytes, we used confocal live imaging and dextran loading of late endosomes/lysosomes [28,29], which are in IFN-γ-activated astrocytes enriched with MHC class II molecules.

Dextran-labeled vesicles moved slower in *GFAP*^*-/-*^*Vim*^*-/-*^ astrocytes than in WT astrocytes, indicating that the lack of IFs does not affect the distribution of late endosomes/lysosomes but reduces their mobility. In WT astrocytes, IFN-γ greatly enhanced the mobility of dextran-labeled vesicles, increasing TL by 36.4% and MD by 69.4%. But in *GFAP*^*-/-*^*Vim*^*-/-*^ astrocytes, the IFN-γ-induced increase was remarkably attenuated. TL increased by only 5.3% and MD by 10.2%. In WT astrocytes, IFN-γ increased the percentage of directionally mobile vesicles, and some vesicles in both treated and control cells exhibited bidirectional mobility [42]. In IFN-γ-activated *GFAP*^*-/-*^*Vim*^*-/-*^ astrocytes, however, the percentage of directionally mobile vesicles increased only slightly. Clearly, IFs help regulate the mobility of MHC class II-positive compartments in IFN-γ-activated astrocytes.

In activated dendritic cells, microtubules drive the directional transport of MHC class II-positive tubules from late endosomes/lysosomes to the plasma membrane [43,44] and their bidirectional movement [14]. Microtubules and actin filaments also help regulate the mobility of MHC class II compartments in antigen-presenting B-cells [11,12] and in a human melanoma cell line (Mel JuSo) [13]. Since IFs in various cell types interact with microfilaments, microtubules, and the motor proteins myosin V, kinesin, and dynein [45,46], the increase in the mobility of MHC class II compartments in IFN-γ-treated astrocytes may reflect up-regulation of IFs [16,47] and the resulting qualitative or quantitative changes in the interaction between IFs and other components of the cytoskeleton. These three cytoskeletal systems may jointly regulate the trafficking and final positioning of MHC class II molecules in IFN-γ-activated astrocytes.

### Proposed (patho)physiological role of IFs in the sorting of MHC class II molecules in IFN-γ-activated astrocytes

Our findings suggest that IFs are involved in the trafficking of MHC class II compartments. Although the lack of IFs attenuated the IFN-γ-induced increase in mobility, MHC class II molecules reached the surface of WT and *GFAP*^*-/-*^*Vim*^*-/-*^ astrocytes. What is the role of IFs in the sorting of MHC class II molecules in IFN-γ-activated astrocytes? We propose that up-regulation of IF protein expression [16,47] in antigen-presenting astrocytes facilitates the transport of antigen-loaded MHC class II molecules from late endosomes/lysosomes in the perinuclear region to the plasma membrane [13] and the recognition of peptide-MHC class II complexes by T-cell receptors on CD4 helper T-cells.

The pathway by which MHC class II molecules reach the surface of APCs is poorly understood [1]. MHC class II-containing compartments may directly fuse with the plasma membrane [43,48]. In astrocytes treated with ATP or glutamate, the fluorescence intensity of lysosomes labeled with FM dye decreases in Ca^2+^-dependent fashion, indicating Ca^2+^-dependent lysosomal exocytosis in these cells [49]. The authors propose that ATP or glutamate stimulation causes the fusion pore to open briefly (kiss-and-run fusion), releasing ATP molecules stored in lysosomes. In astrocytes, stronger and sustained elevation of [Ca^2+^_i_ induced with Ca^2+^ ionophores or by mechanical stimulation triggers predominantly full fusion of lysosomes with the plasma membrane [31,49]. Both modes of lysosomal exocytosis could deliver lysosomal membrane-bound MHC class II molecules to the plasma membrane [50,51].

Although ATP did not induce enhanced release of labeled dextran from late endosomes/lysosomes, which would indicate lysosomal fusion [28,30,31], it is possible that astrocytes use the lysosomal exocytotic machinery [52] to deliver MHC class II molecules to the plasma membrane. In lysosomes, the increase in [Ca^2+^_i_ in response to ATP stimulation is delayed, usually by several minutes [30,49]. Since we tracked the mobility of dextran-labeled vesicles for 30 s after application of ATP, we could not detect additional lysosomal fusion events. However, when we treated astrocytes with 1 μM adrenalin we did observe the release of labeled dextran from late endosomes/lysosomes (unpublished data), implying a role of cAMP in lysosomal exocytosis. ATP stimulation reduced the mobility of late endosomes/lysosomes. In non-secretory cells, the fraction of mobile lysosomes is reduced after the addition of a Ca^2+^ ionophore, and in astrocytes, the fraction of Lysotracker-labeled vesicles containing atrial natriuretic peptide is reduced after stimulation with ATP and Ca^2+^ ionophore [20,53].

The ATP-induced attenuation of vesicle mobility was more apparent in the presence of IFs (Figure 5), implying a role for IFs in this process [20]. Reduced lysosomal mobility may increase the probability that lysosomes will dock and fuse with the plasma membrane. Thus, in astrocytes functioning as APCs, an increase in [Ca^2+^_i_ and cAMP (unpublished data) may trigger the delivery of antigen-loaded MHC class II molecules from the lysosomal membrane to the plasma membrane.

## Conclusions

The immune privilege of the CNS makes it crucial that the activation of the immune system by resident immunocompetent astrocytes is strictly regulated. We show here that IFN-γ induces the expression of MHC class II molecules in otherwise immunologically silent astrocytes and increases the mobility of vesicles enriched with MHC class II molecules. The increase is highly dependent on IFs, suggesting that the up-regulation of IFs in reactive astrocytes allows faster and therefore more efficient delivery of MHC class II-positive compartments to the cell surface and the activation of CD4 helper T-cells. Our data also suggest that besides IFN-γ activation, astrocytes have a regulatory mechanism, involving increases in [Ca^2+^]_i_, and cAMP that controls the fusion of MHC class II compartments with the plasma membrane and the final delivery of MHC class II molecules to the cell surface. In vivo, such regulatory mechanisms may enable antigen-presenting astrocytes to respond rapidly and in a controlled manner to CNS inflammation. For better understanding of the role of astrocytes in CNS pathologies, it will be essential to reveal in greater detail the immunological function of astrocytes and the complex regulatory mechanisms that enable these cells to mediate immune responses in the CNS.

## Abbreviations

APCs, Antigen-presenting cells; CNS, Central nervous system; GFAP, Glial fibrillary acidic protein; IF, Intermediate filament; LAMP1, Lysosome-associated membrane protein 1; MD, Maximal displacement; MHC, Major histocompatibility complex; TL, Track length; Vim, Vimentin; WT, Wild-type.

## Competing interests

The authors declare that they have no competing financial interests.

## Authors’ contributions

NV and MG designed, performed the experiments, analyzed the data, and prepared the figures. MPo designed and performed the experiments. UŠ and MJ carried out flow cytometry study. YP, MF, and MPe generated *GFAP*^*-/-*^*Vim*^*-/-*^ and WT mice and astrocytes. MK helped with data analysis. NV and RZ wrote the paper. RZ conceived and supervised the experiments. All of the authors discussed the results and commented on the manuscript. All authors read and approved the final version of the manuscript.

## Supplementary Material

Additional file 1 **Mobility of dextran labeled vesicles in WT astrocytes.** The tracks of individual vesicles were monitored for 30 s. Click here for file

Additional file 2 **Mobility of dextran labeled vesicles in IFN-γ activated WT astrocytes.** The tracks of individual vesicles were monitored for 30 s. (AVI 595 kb) Click here for file

Additional file 3 **Mobility of dextran labeled vesicles in*GFAP***^***-/-***^***Vim***^***-/-***^**astrocytes.** The tracks of individual vesicles were monitored for 30 s. (AVI 993 kb) Click here for file

Additional file 4 **Mobility of dextran labeled vesicles in IFN-γ activated*GFAP***^***-/-***^***Vim***^***-/-***^**astrocytes.** The tracks of individual vesicles were monitored for 30 s. (AVI 578 kb) Click here for file

Additional file 5 **Mobility of dextran labeled vesicles in WT astrocytes upon ATP stimulation.** The tracks of individual vesicles were monitored for 30 s. Click here for file

Additional file 6 **Mobility of dextran labeled vesicles in IFN-γ activated WT astrocytes upon ATP stimulation.**The tracks of individual vesicles were monitored for 30 s. Click here for file

Additional file 7 **Mobility of dextran labeled vesicles in*GFAP***^***-/-***^***Vim***^***-/-***^**astrocytes upon ATP stimulation.**The tracks of individual vesicles were monitored for 30 s. Click here for file

Additional file 8 **Mobility of dextran labeled vesicles in IFN-γ activated*GFAP***^***-/-***^***Vim***^***-/-***^**astrocytes upon ATP stimulation.**The tracks of individual vesicles were monitored for 30 s. Click here for file
